# Genetic variations and dog breed identification using inter-simple sequence repeat markers coupled with high resolution melting analysis

**DOI:** 10.7717/peerj.10215

**Published:** 2020-10-30

**Authors:** Wannapimol Kriangwanich, Korakot Nganvongpanit, Kittisak Buddhachat, Puntita Siengdee, Siriwadee Chomdej, Siriluck Ponsuksili, Chatchote Thitaram

**Affiliations:** 1 Department of Veterinary Biosciences and Public Health, Faculty of Veterinary Medicine, Chiang Mai University, Chiang Mai, Thailand; 2 Excellence Center in Veterinary Bioscience, Chiang Mai University, Chiang Mai, Thailand; 3 Department of Biology, Faculty of Science, Naresuan University, Phitsanulok, Thailand; 4 Leibniz Institute for Farm Animal Biology, Dummerstorf, Germany; 5 Department of Biology, Faculty of Science, Chiang Mai University, Chiang Mai, Thailand; 6 Center of Excellence in Elephant and Wildlife Research, Faculty of Veterinary Medicine, Chiang Mai University, Chiang Mai, Thailand

**Keywords:** Breeds, Canine, Classification, Melting temperature, Genetic variation

## Abstract

The identification of differing physical characteristics of dogs is an uncomplicated and straightforward way to categorize dog breeds. However, many dog owners and veterinarians still struggle to distinguish between pure breed and mixed variations in certain breeds of dogs. Presently, the absence of the tools and methods needed to confirm a pure breed dog is a significant problem since the only method available to validate pure or mongrel breeds is the official pedigree system. Inter-simple sequence repeat markers have been successfully used to assess genetic variations and differentiations. Notably, inter-simple sequence repeat markers coupled with high resolution melting analysis were effectively used for the breed identification of 43 breeds of dogs (total 463 dogs). The 10 primers chosen for analysis resulted in a range of 31–78.6% of breed discrimination when using one primer, while a combination of two primers was able to successfully discriminate between all of the 43 dog breeds (100%). Shannon’s index information (*I* = 2.586 ± 0.034) and expected heterozygosity (*H*_*e*_ = 0.908 ± 0.003) indicated a high level of genetic diversity among breeds. The fixation index (*F*_*st*_) revealed a value of 10.4%, demonstrating that there was a high level of genetic subdivision between populations. This study showed that inter-simple sequence repeat marker analysis was effective in demonstrating high genetic diversity among varying breeds of dogs, while a combination of Inter-simple sequence repeat marker analysis and high resolution melting analysis could provide an optional technique for researchers to effectively identify breeds through genetic variations.

## Introduction

More than 400 dog breeds have been registered with Federation Cynologique Internationale (FCI) and other official kennel clubs (Bigi et al., 2015; Bjornerfeldt et al., 2008; Parker et al., 2004; Suarez et al., 2012). However, many of the more recently registered breeds have been developed from existing breeds and have subsequently been established as new breeds with particular phenotypic traits (Irion et al., 2003). Breeding programs that generate specific morphologies are a major problem and can lead to inherited disorders among purebred dogs (Parker et al., 2004). The mating process between two animals or breeds with relatively close lines of ancestry may increase the proportion of homozygotes in all loci. This can also occur as a result of the practice of human intensive selection or the intentional breeding of selected dog breeds with limited gene pools. In addition, if deleterious recessive alleles are abundant, it may enhance the risk of genetic disorders as well (Koskinen & Bredbacka, 2000). Backcrossing and inbreeding have been used to fix certain characteristics and traits that lead to decreases in heterozygosity levels. Yet, some of these undesirable traits may have resulted from rapid phenotypic selection wherein each breed was generated from multiple origins with a historical gene flow (Bigi et al., 2015; Boldura et al., 2017; Koskinen & Bredbacka, 2000; Parra et al., 2008; Shinkarenko et al., 2010). Likewise, genetic diversity, differentiation and the genetic structure of different dog breeds have routinely been evaluated based on mitochondrial DNA (Suarez et al., 2012), single nucleotide polymorphism (SNPs) (Boyko et al., 2010; Shannon et al., 2015) and nuclear microsatellites (Bigi et al., 2018; Bigi et al., 2015; Irion et al., 2003; Irion et al., 2005; Kim et al., 2001; Lee, Choi & Cho, 2014; Mellanby et al., 2013; Parker et al., 2004; Pedersen et al., 2013; Schelling, Gaillard & Dolf, 2005; Shinkarenko et al., 2010).

Genetic polymorphism can be similar to other types of biological polymorphism as it occurs when members of species differ in form or shape. Polymorphism can be associated with one or more nucleotide changes and variations in DNA sequences that occur within a population at a frequency of 1% or higher. The higher incidence in a given population suggests that a polymorphism is naturally occurring (Brookes, 1999; Karki et al., 2015; Richards et al., 2015). Several molecular markers have been used to estimate the genetic diversity of members of the same species (Boldura et al., 2017). A good marker is one with high genetic variability and has the ability to generate multi-locus data (Anne, 2006). Inter-simple sequence repeat (ISSR) are comprised of segments of DNA that are flanked by microsatellite at both ends. With this method there is no requirement for the prior DNA information of a studied species. As such, the ISSR markers located in neutral genomic regions could be more diverse and reflect the recent dynamic events of a given population such as variations in effective population size, as well as incidences of bottle necks and migration (Steffler et al., 2016). Because ISSR is a multi-locus technique, the main limitations of ISSR markers are that there can be dominant markers that cannot separate heterozygotes. High resolution melting analysis (HRM) coupled with PCR (PCR-HRM) requires the use of fluorescent dye to detect signals from any perceived differences in nucleotide length and composition, which can reveal results at different melting temperatures (Erali & Wittwer, 2010; Reed, Kent & Wittwer, 2007; Vossen et al., 2009; Wittwer et al., 2003). Several studies have reported on the successful identification of plant and bacteria species using HRM with DNA fingerprinting including *Phyllanthus amarus* (Tulsiani et al., 2010) and *Leptospira spp.* (Buddhachat, Changtor & Ninket, 2019; Naze et al., 2015). Melting profiles can be measured in real time using the universal ISSR primer combined with specific software that can be employed to explore and quantify them in order to reduce the subjective errors that are associated with human biases (Power, 1996; Tulsiani et al., 2010).

The outcomes of this study have been divided into two parts in order to fulfil the objectives of the study. First, we aimed to assess the genetic diversity of different dog breeds that inhabit northern Thailand in order to provide information on genetic variations and differentiations. The second objective was to develop a selective approach that uses DNA fingerprinting coupled with HRM to identify the breed of a dog.

## Materials and Methods

### Sample collection

A total of 463 domestic dogs from 43 different breeds that were not related were selected for the collection of one mL-blood samples. This blood was obtained from a cephalic or saphenous vein. The samples were then transferred into 3-mL ethylenediaminetetraacetate tubes (EDTA) by veterinarians at small animal hospitals located in northern Thailand (Table 1). Varying breeds of dogs were recorded according to statements made by qualified veterinarians who applied FCI nomenclature standards. Each breed was then sub-divided into eight groups as recognized by the FCI classification protocol with the exceptions of the American Bully and the American Pit Bull Terrier. These two breeds do not belong to any specified groups according to FCI classification. The provisions for ethical regulations were approved of by the Animal Use Committee of the Faculty of Veterinary Medicine, Chiang Mai University, Thailand in 2018 (S32/2561).

### DNA extraction

DNA were extracted from whole blood samples by using a RBC Bioscience™ Real genomics DNA extraction kit for Blood/Bacteria/Cultured cells (RBC Bioscience Corp., New Taipei, Taiwan) following the manufacturer’s instructions with the DNA final volume that was recovered by reckoning a final 50 µl of a standard elution. Lastly, Beckman Coulter DU^^®^^ 730 spectrophotometer (Beckman Coulter, CA, USA) was used for determining the The concentration, yield, and purity of DNA and was preserved under −20 °C for further analysis.

### Genetic variations and genetic differentiation assessment

Thirty-four ISSR primers from The University of British Columbia primer set of microsatellite (Microsatellite UBC primer set 9, University of British Columbia, Vancouver, Canada) were screened by polymerase chain reaction (PCR) technique and performed in this study following our previously protocol (Kriangwanich et al., 2018). Briefly, 43 samples were amplified individually by PCR which consisted of Buffers, dNTP, ISSR primer and *Taq* DNA polymerase with 10 ng of DNA template. Finally, deionized water was added to a volume of 25 µl and also served as a negative control. PTC-200 at DNA EngineThermal Cycler (Bio-Rad Laboratories, Inc., CA, USA) was used for PCR amplifications under the condition from our previous study (Kriangwanich et al., 2018). The PCR products were stained and separated electrophoretically on 2% agarose gel by PowerPac 200 (Bio-Rad, CA, USA) containing 1X Tris-acetate-ethylenediaminetetraacetate (TAE) buffer at 120 V for 30 min and visualized under UV light by using GelMax 125 Imager (UVP, Cambridge, England).

## Statistical analysis

The DNA fingerprints of 43 dog breeds obtained from ISSR-HRM using the screened ISSR primer were used to score clearly observed bands that served as binary symbols, wherein the presence of a band was 1 and the absence of a band was 0 (Fig. S1). The binary matrix that was created allowed us to determine the level of polymorphism for each primer as represented by the polymorphic percentage using the formula of Ng & Tan (2015). In this study, The inter-population genetic variations was measured by a diversity index including the observed number of alleles (N_a_), the effective number of alleles (N_e_), and the expected level of heterozygosity within the population (H_e_). Shannon’s information index (I). Fixation index within each breed (*F*_is_) and among breeds (*F*_st_), along with the parameter of the gene flow (N_m_), were assessed to measure the degree of population differentiation from the genetic structure. Furthermore, the genetic similarity and genetic distance of domestic dogs from each breed and between breeds were calculated using the method described by Takezaki & Nei (2008). All parameters mentioned above were analyzed through GenAlEx version 6.503 program with Microsoft Excel (Peakall & Smouse, 2006). The genetic similarity and genetic distance values were calculated using pairwise comparisons of domestic dog breeds. Bichon Frise, Boston Terrier, Chow Chow, Welsh Corgi, Dachshund, Dalmatian, Doberman Pishcher, Pekingese, Thai Ridgeback, Alaskan Malamute, Cavalier King Charles Spaniel, West Highland White Terrier, Akita Inu, Border Collie, Dogo Argentino, Fila Brasileiro, Great Dane, Papillon and Shiba Inu breeds were excluded from the genetic variation assessment and STURCTUE program because the number of samples was less than 4; however, they will later be included in the ISSR-HRM analysis. Determination of the population structure and any distinct genetic characteristics among populations of domestic dogs was done by STRUCTURE (Pritchard, Stephens & Donnelly, 2000). All analyses were run with a burn-in period of 100,000. Additionally, 500,000 repetitions were employed three times in order to validate the resulting outcomes. In addition, DARwin software program version 6.0 (Perrier, Flori & Bonnot, 2003) and the interactive tree of life web-based program (Letunic & Bork, 2019) were used to create dendrograms of dog breeds based on the unweighted neighbor-joining method.

**Table 1:** Number of samples of 43 dog breeds classified by Federation Cynologique Internationale (FCI) breeds nomenclature.

Breeds	FCI group	Numbers
German Shepherd	1	22
Welsh Corgi	1	3
Border Collie	1	1
Miniature Pinscher	2	6
Schnauzer	2	6
Bulldog	2	5
Rottweiler	2	5
Doberman Pinscher	2	3
Dogo Argentino	2	1
Fila Brasileiro	2	1
Great Dane	2	1
Yorkshire Terrier	3	16
Jack Russell Terrier	3	10
Bull Terrier	3	4
West Highland White Terrier	3	2
Dachshund	4	3
Pomeranian	5	47
Siberian Husky	5	31
Thai Bangkaew	5	18
Samoyed	5	6
Spitz	5	6
Chow Chow	5	3
Thai Ridgeback	5	3
Alaskan Malamute	5	2
Akita Inu	5	1
Shiba Inu	5	1
Beagle	6	28
Dalmatian	6	3
Golden Retriever	8	40
Labrador Retriever	8	14
Cocker Spaniel	8	5
Chihuahua	9	46
Shih Tzu	9	37
French Bulldog	9	29
Poodle	9	17
Pug	9	11
Bichon Frise	9	3
Boston Terrier	9	3
Pekingese	9	3
Cavalier King Charles Spaniel	9	2
Papillon	9	1
American Bully	–	9
American Pit Bull Terrier	–	5
Total		463

**Notes.**

* 1 = Sheepdogs and Cattledogs; 2 = Pinscher and Schnauzer-Molossoid and Swiss Mountain and Cattledogs; 3 = Terriers; 4 = Dachshunds; 5 = Spitz and primitive types; 6 = Scent hounds and related breeds; 8 = Retrievers-Flushing dogs-Water dogs; 9 = Companion and Toy dogs.

### Breeds identification by ISSR-HRM

#### Primer screening

From the preliminary primer screening process using PCR, there were 13 primers (Table 2) that yielded a reproducible DNA fingerprint. In this experiment, these primers were adopted to generate a melting profile of the DNA fingerprints based on the ISSR fingerprints established by HRM, or what is referred to as “ISSR-HRM”. The suitable ISSR primers were used to create melting fingerprints using ISSR-HRM for 463 individual subjects from 43 breeds. ISSR-HRM was carried out at a final volume of 10 µl containing 1X SensiFast™ HRM kit (EvaGreen^®^ dye, dNTPs and enhancers) (Bioline, TN, USA), 0.5 µM ISSR primer and a 10 ng DNA template. Deionized water was added instead of the DNA template to establish a negative control.

**Table 2:** Nucleotide sequences of Inter-simple sequence repeat (ISSR) primers obtained from University of British Columbia.

Primers	Sequence (5′–3′)	Length
UBC809	AGA GAG AGA GAG AGA GG	17-mer
UBC817	CAC ACA CAC ACA CAC AA	17-mer
UBC818	CAC ACA CAC ACA CAC AG	17-mer
UBC823	TCT CTC TCT CTC TCT CC	17-mer
UBC825	ACA CAC ACA CAC ACA CT	17-mer
UBC826	ACA CAC ACA CAC ACA CC	17-mer
UBC827	ACA CAC ACA CAC ACA CG	17-mer
UBC835	AGA GAG AGA GAG AGA GYC	18-mer
UBC844	CTC TCT CTC TCT CTC TRC	18-mer
UBC847	CAC ACA CAC ACA CAC ARC	18-mer
UBC848	CAC ACA CAC ACA CAC ARG	18-mer
UBC861	ACC ACC ACC ACC ACC ACC	18-mer
UBC866	CTC CTC CTC CTC CTC CTC	18-mer

#### ISSR-HRM

First, ISSR-HRM was performed on a PCRmax Eco 48 machine (PCRmax limited, Staffordshire, UK). The initial step was initiated at 95 °C for 10 min followed by 37 cycles of the denaturation step at 95 °C for 20 s. This was followed by annealing with an extension step at 58 °C for 45 s. During the amplification process, fluorescence data were collected at the end of each annealing cycle. HRM analysis was carried out after 37 real-time PCR cycles at temperature increments of 0.1 °C/cycle between values of 55 °C and 95 °C in order to generate high resolution melting curves. Melt curve profiles from ISSR-HRM, normalized curves, difference curves and amplification curves were all generated using Eco software v5.2.12 (PCRmax) to identify any differentiations between the melting profiles of 43 dog breeds. Secondly, the amplicons obtained from ISSR-HRM were stained and separated electrophoretically on 2% agarose gel according to Kriangwanich et al. (2018). Finally, the pre- and post-melt normalization regions were set to define temperature boundaries and generate the normalization melting curves. Melting Temperature (Tm) and Melting pattern were recorded in order to calculate the percent of discrimination and to create an accurate heatmap.

## Results

### ISSR Polymorphism

We screened 34 ISSR primers under five different annealing temperature conditions (55, 56, 57, 58 and 59 °C). It was found that there were 13 primers of UBC set at 9 and an annealing temperature of 58 °C providing reproducible and informative results from 43 dog breeds. Thus, 13 ISSR primers were reassessed to generate ISSR fingerprints by real-time PCR followed by HRM analysis (ISSR-HRM). The results revealed that 10 out of 13 primers at an annealing temperature of 58 °C were enabled to generate melting curves for all 43 dog breeds.

**Figure 1:**
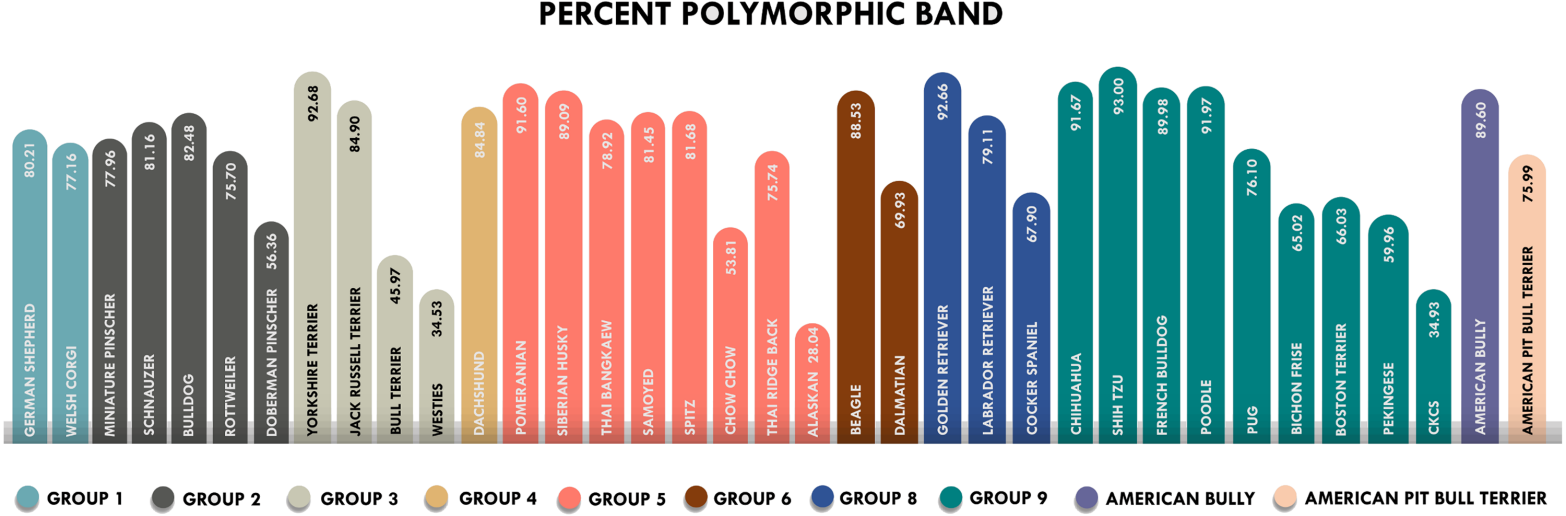
Percent polymorphic bands of 36 breeds of dogs classified by FCI groups. Each color represents each group. The highest percent of polymorphic bands was 93% in the Shih Tzu breed and the lowest was 28.04% in the Alaskan Malamute. *Westies = West Highland White Terrier, Alaskan = Alanskan Malamute and CKCs = Cavalier King Charles Spaniel.

The amplicons obtained from ISSR-HRM were subjected to agarose gel electrophoresis to investigate the DNA fingerprints. Our findings indicate that the percent polymorphic bands ranged from the lowest polymorphism at 28.04% in the Alaskan malamute breed to the highest polymorphism at 93% in the Shih Tzu breed (Fig. 1). The breeds of Bull Terrier, Cavalier King Charles Spaniels, Alaskan Malamute and West Highland White Terrier all indicated low polymorphism values of less than 50%. The Shih Tzu, Pomeranian, Chihuahua, Golden Retriever, Poodle and Yorkshire Terrier breeds all displayed very high polymorphism values at more than 90%, indicating substantial genetic diversity at the population level.

### Genetic variations and differentiation among different dog breeds

Number of bands and band size ranges are shown in File S1. The number of bands established from the 10 ISSR primers ranged from 3,771 bands from primer UBC823 to 6,552 bands from primer UBC848 obtained from 43 dog breeds. Genetic variations of each dog breed are shown in Table 3. The observed number of alleles (N_a_) ranged from 7.900 in the Bull Terrier breed to 36.500 in the Pomeranian breed. Values of the effective number of alleles (N_e_) were similar to N_a_ in the Miniature Pinscher, Schnauzer, Bulldog, Cocker Spaniel, American Pit Bull Terrier and Rottweiler breeds. Meanwhile, N_e_ values in the Pomeranian, Chihuahua, Golden Retriever, Shih Tzu, Siberian Husky, French Bulldog, Beagle, German Shepherd, Thai Bangkaew, Poodle, Yorkshire Terrier, Labrador Retriever, Pug, Jack Russell Terrier, American Bully, Samoyed, Spitz and Bull Terrier breeds were less than the values of N_a_. According to Shannon’s information index (I), the Pomeranian breed recorded the highest value, while the Bull Terrier breed presented the lowest value. A high value of heterozygosity indicates considerable outbreeding. The mean expected heterozygosity (H_e_) value for the overall populations was 0.831 ± 0.008, of which the Pomeranian and Siberian Husky breeds were identified as the most diverse groups while the Bull Terrier breed displayed the lowest degree of heterozygosity. This outcome was in accordance with the values of N_a_, N_e_ obtained from Shannon’s information index (I). In this study, negative *F*_is_ and *F*_it_ values were observed from all of the ISSR markers (Table 4).

The fixation index test was used to estimate the degree of genetic differentiation of each dog breed in this study. The maximum fixation index (*F*_st_) value was found among the Pomeranian population, and the minimum value was found in the Bull Terrier population (Table 3). Multilocus *F*_st_ values indicated that 15% of the total genetic variations was explained by breed differences with the remaining 85% corresponding to differences among individual dogs. Apart from the *F*_st_ value, gene flow is an important mechanism for transferring genetic diversity among populations and is represented by the N_m_ values. The results indicated that the N_m_ values were more than one for every ISSR marker, for which UBC823 reported the highest value of N_m_ (Table 4). In addition, the relative genetic distances among breeds were examined by calculating the mean pairwise *F*_st_ values for each breed (File S2). A high *F*_st_ value between two populations indicated that they were reproductively separate. The most divergent breeds were Chow Chow and Boston Terrier. The least distinct group was Yorkshire Terrier and Siberian Husky, followed by the French Bulldog and the Poodle groups. The genetic distance was also determined by Nei’s genetic distance analysis (Table S1). Nei’s genetic distance analysis indicated that the closest distance was obtained between the Yorkshire Terrier and Siberian Husky breeds, which was similar to the pairwise *F*_st_ value. Additionally, the greatest value was recorded between the Welsh Corgi and Bull Terrier breeds.

**Table 3:** Diversity indices for 10 inter-simple sequence repeat (ISSR) markers evaluated on different dog breeds.

Pop	N_a_ ± SE	N_e_ ± SE	I ± SE	H_e_ ± SE	*F*_st_ ± SE
German Shepherd	27.300 ± 1.391	23.136 ± 1.491	3.203 ± 0.064	0.955 ± 0.004	0.048 ± 0.004
Miniature Pinscher	12.000 ± 0.000	12.000 ± 0.000	2.485 ± 0.000	0.917 ± 0.000	0.091 ± 0.000
Schnauzer	12.000 ± 0.000	12.000 ± 0.000	2.485 ± 0.000	0.917 ± 0.000	0.091 ± 0.000
Bulldog	10.000 ± 0.000	10.000 ± 0.000	2.303 ± 0.000	0.900 ± 0.000	0.111 ± 0.000
Rottweiler	10.000 ± 0.000	10.000 ± 0.000	2.303 ± 0.000	0.900 ± 0.000	0.111 ± 0.000
Yorkshire Terrier	28.700 ± 0.761	26.890 ± 1.025	3.323 ± 0.033	0.962 ± 0.001	0.039 ± 0.002
Jack Russell Terrier	19.000 ± 0.683	18.605 ± 0.949	2.924 ± 0.050	0.945 ± 0.004	0.059 ± 0.004
Bull Terrier	7.900 ± 0.100	7.840 ± 0.160	2.062 ± 0.017	0.872 ± 0.003	0.147 ± 0.004
Pomeranian	36.500 ± 2.432	28.575 ± 2.019	3.442 ± 0.067	0.963 ± 0.003	0.038 ± 0.003
Siberian Husky	32.200 ± 1.298	27.458 ± 1.035	3.381 ± 0.039	0.963 ± 0.001	0.038 ± 0.001
Thai Bangkaew	26.000 ± 1.317	22.845 ± 1.314	3.178 ± 0.062	0.954 ± 0.003	0.048 ± 0.004
Samoyed	11.700 ± 0.213	11.629 ± 0.249	2.455 ± 0.020	0.914 ± 0.002	0.095 ± 0.002
Spitz	11.900 ± 0.100	11.829 ± 0.171	2.473 ± 0.012	0.915 ± 0.001	0.074 ± 0.017
Beagle	30.000 ± 1.491	24.923 ± 1.484	3.289 ± 0.054	0.959 ± 0.002	0.043 ± 0.003
Golden Retriever	32.400 ± 1.137	25.288 ± 1.087	3.335 ± 0.040	0.960 ± 0.002	0.042 ± 0.002
Labrador Retriever	24.700 ± 0.895	22.929 ± 1.264	3.163 ± 0.047	0.955 ± 0.003	0.047 ± 0.003
Cocker Spaniel	10.000 ± 0.000	10.000 ± 0.000	2.303 ± 0.000	0.900 ± 0.000	0.111 ± 0.000
Chihuahua	25.700 ± 1.814	19.986 ± 1.368	3.086 ± 0.065	0.948 ± 0.003	0.055 ± 0.004
Shih Tzu	29.800 ± 1.218	24.380 ± 1.100	3.276 ± 0.044	0.958 ± 0.002	0.044 ± 0.002
French Bulldog	32.900 ± 1.433	27.395 ± 1.461	3.382 ± 0.062	0.962 ± 0.003	0.040 ± 0.004
Poodle	28.900 ± 1.286	26.780 ± 1.397	3.316 ± 0.057	0.961 ± 0.003	0.040 ± 0.003
Pug	19.200 ± 0.827	18.005 ± 1.038	2.912 ± 0.054	0.943 ± 0.004	0.061 ± 0.004
American Bully	17.300 ± 0.335	17.062 ± 0.421	2.843 ± 0.022	0.941 ± 0.002	0.063 ± 0.002
American Pit Bull Terrier	10.000 ± 0.000	10.000 ± 0.000	2.303 ± 0.000	0.900 ± 0.000	0.111 ± 0.000
Total	16.973 ± 0.588	15.259 ± 0.475	2.586 ± 0.034	0.908 ± 0.003	0.104 ± 0.004

**Table 4:** Fixation indices and gene flow parameters for inter-simple sequence repeat (ISSR) markers evaluated on different dog breeds.

Primers	*F*_is_	*F*_it_	*F*_st_	N_m_
UBC809	−0.203	−0.022	0.151	1.404
UBC817	−0.203	−0.020	0.152	1.391
UBC818	−0.202	−0.021	0.151	1.408
UBC823	−0.210	−0.033	0.146	1.458
UBC825	−0.204	−0.021	0.152	1.398
UBC826	−0.205	−0.024	0.150	1.417
UBC827	−0.202	−0.022	0.149	1.423
UBC835	−0.203	−0.021	0.151	1.405
UBC844	−0.203	−0.024	0.148	1.436
UBC848	−0.201	−0.019	0.152	1.400
Mean	−0.204	−0.023	0.150	1.414

### Population structure and phylogenetic dendrograms

**Figure 2:**
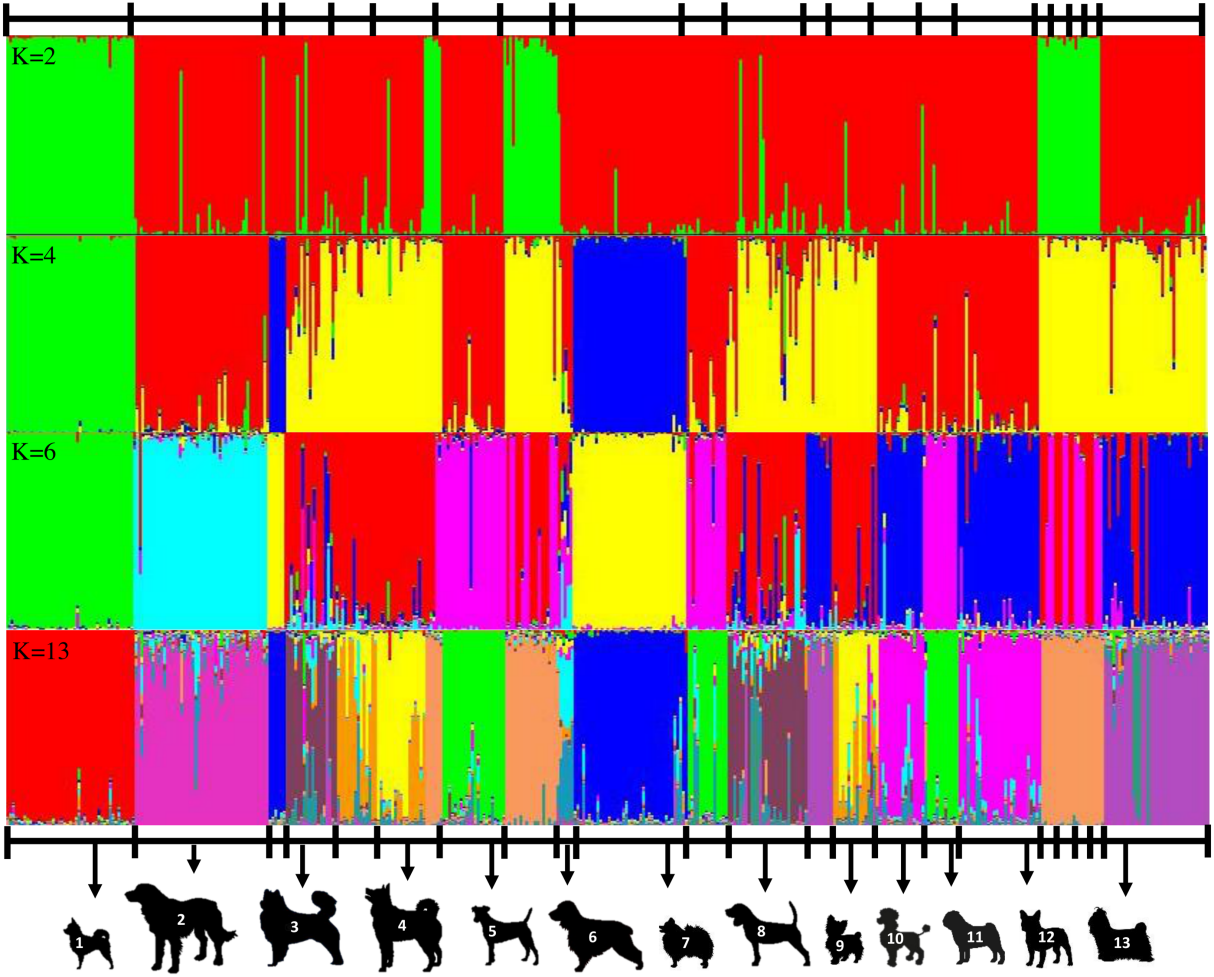
Clustering assignment of 22 dog breeds based on data of 10 ISSR makers. STRUCTURE was used to determine the admixture of each dog. Each breed is represented by 4-47 animals at K values (number of genetic clusters) from 2 to the most suitable K at 13. Each vertical line represents an individual dog. A clear indication of two subgroups was obtained at all K values were at 2 and four subgroups had a K value of 4. The figure shows representative runs at K = 2, K = 4, K = 6 and K = 13 and labels 13 distinctive breeds as 1 = Chihuahua, 2 = Golden Retriever, 3 = Thai Bangkaew, 4 = Siberian Husky, 5 = Jack Russell Terrier, 6 = Cocker Spaniel, 7 = Pomeranian, 8 = Beagle, 9 = Yorkshire Terrier, 10 = Poodle, 11 = Pug, 12 = French Bulldog, 13 = Shih Tzu.

**Figure 3:**
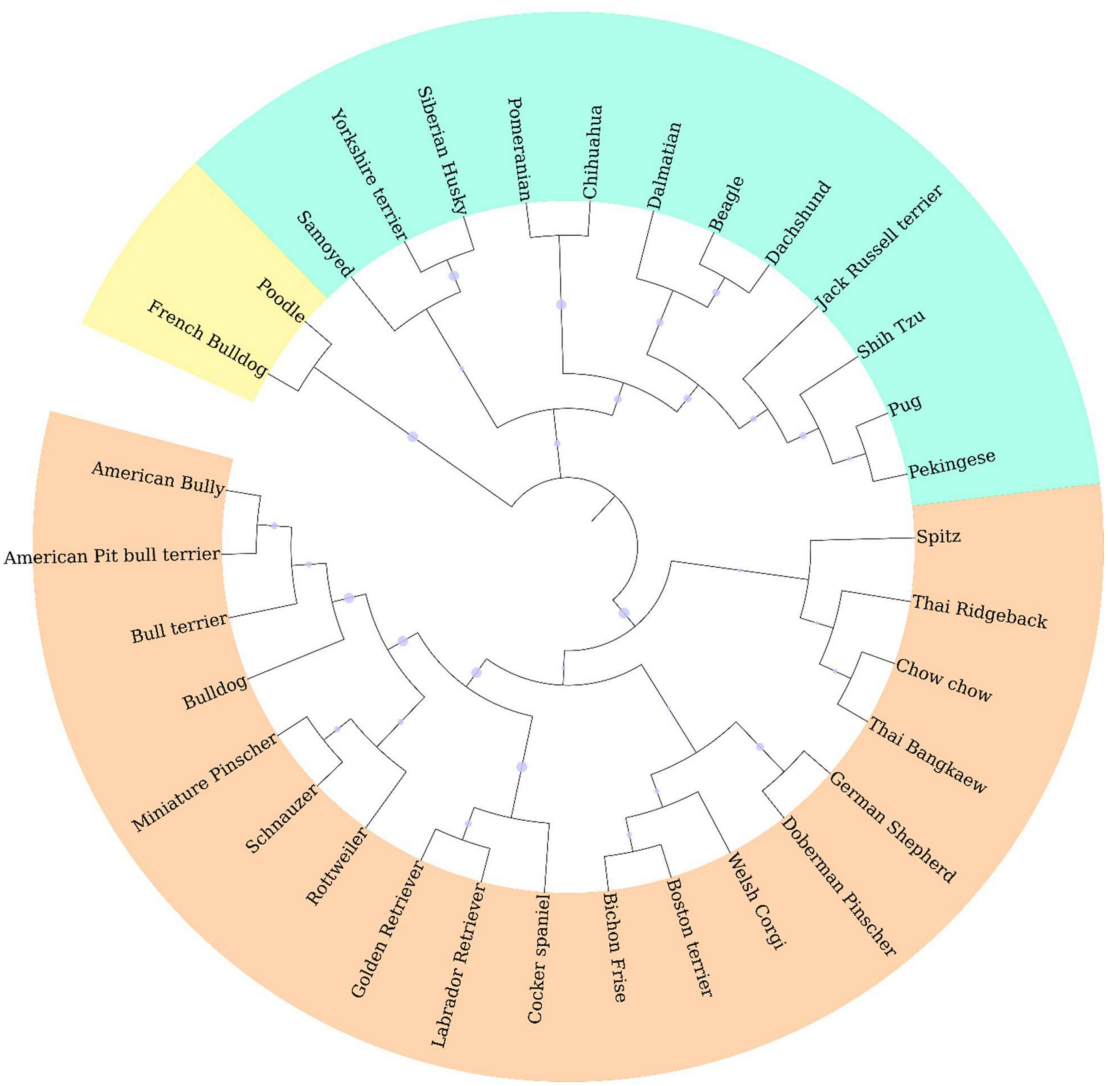
Phylogenetic dendrogram of 33 different dog breeds separated into three main clusters based on genetic distance which is presented in three different colors.

Genotypic information derived from 10 ISSR markers was analyzed by the STRUCTURE program which was employed using increasing values of subpopulations (K). At *K* = 2, one cluster was anchored by eight breeds, whereas the other clusters contained large numbers of other breeds (Fig. 2A). The results from the STRUCTURE revealed that there were differentiations between breeds at *K* = 13 (Fig. 2D). The Cocker Spaniel group was the most diverse group followed by the Beagle and Thai Bangkaew groups, while the contribution of the Chihuahua, Jack Russell Terrier, Pug and Golden Retriever groups were found to be the most homogeneous. STRUCTURE analysis also stipulated that some individuals had been assigned to the wrong group or breed. For example, there were non-Shih Tzu or non-Labrador Retriever breeds in the study that were inaccurately assigned to the Shih Tzu and Labrador Retriever groups. Moreover, the Beagle, Thai Bangkaew, Yorkshire Terrier and Siberian Husky groups represented a less than 50% contribution of the dominant subgroup as was observed among the other members of these breeds. A phylogenetic tree of 33 dog breeds was used to separate the dog breeds into 3 main clusters (Fig. 3). The Asian Spitz breed was grouped together with the Spitz split and the Thai Ridgeback. The Thai Bangkaew and Chow Chow breeds were grouped together along with Nordic Sledge dogs. Additionally, Sheep-dogs and Cattle-dogs, Pinschers, Retrievers and Scent Hounds were all grouped in the same cluster. The American Pit Bull Terrier and American Bully breeds were grouped with the Bull Terrier and Bulldog breeds, while the French Bulldog and Poodle were grouped in the same cluster but were separated from all other clusters. On the other hand, companion and toy dogs were classified in a number of different clusters.

### Melting profile

**Figure 4:**
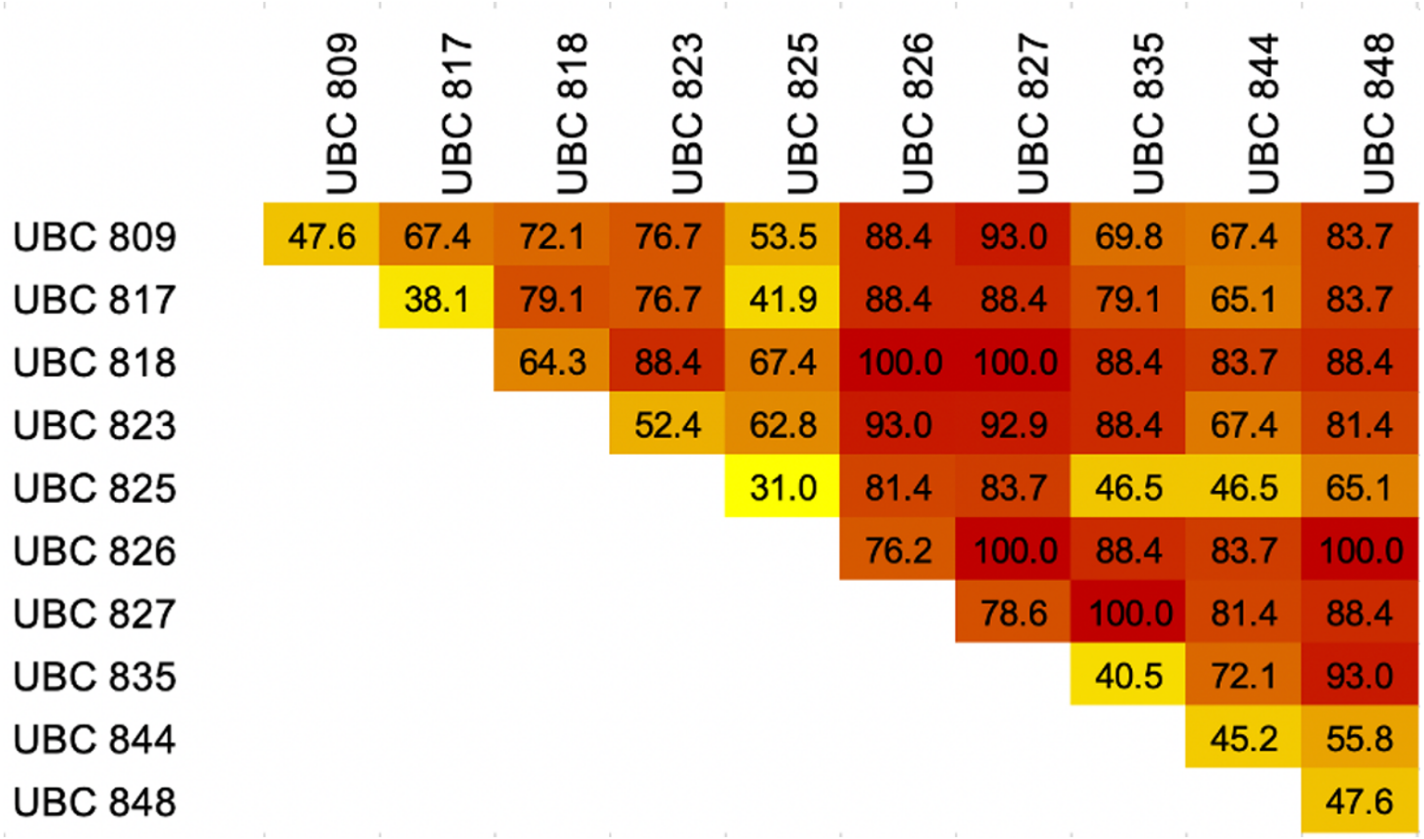
Heatmap showing percentages of breed discrimination from each breed and combination of Inter-simple sequence repeat (ISSR) markers coupled with High Resolution melting analysis (HRM) for identification of dog breeds.

**Figure 5:**
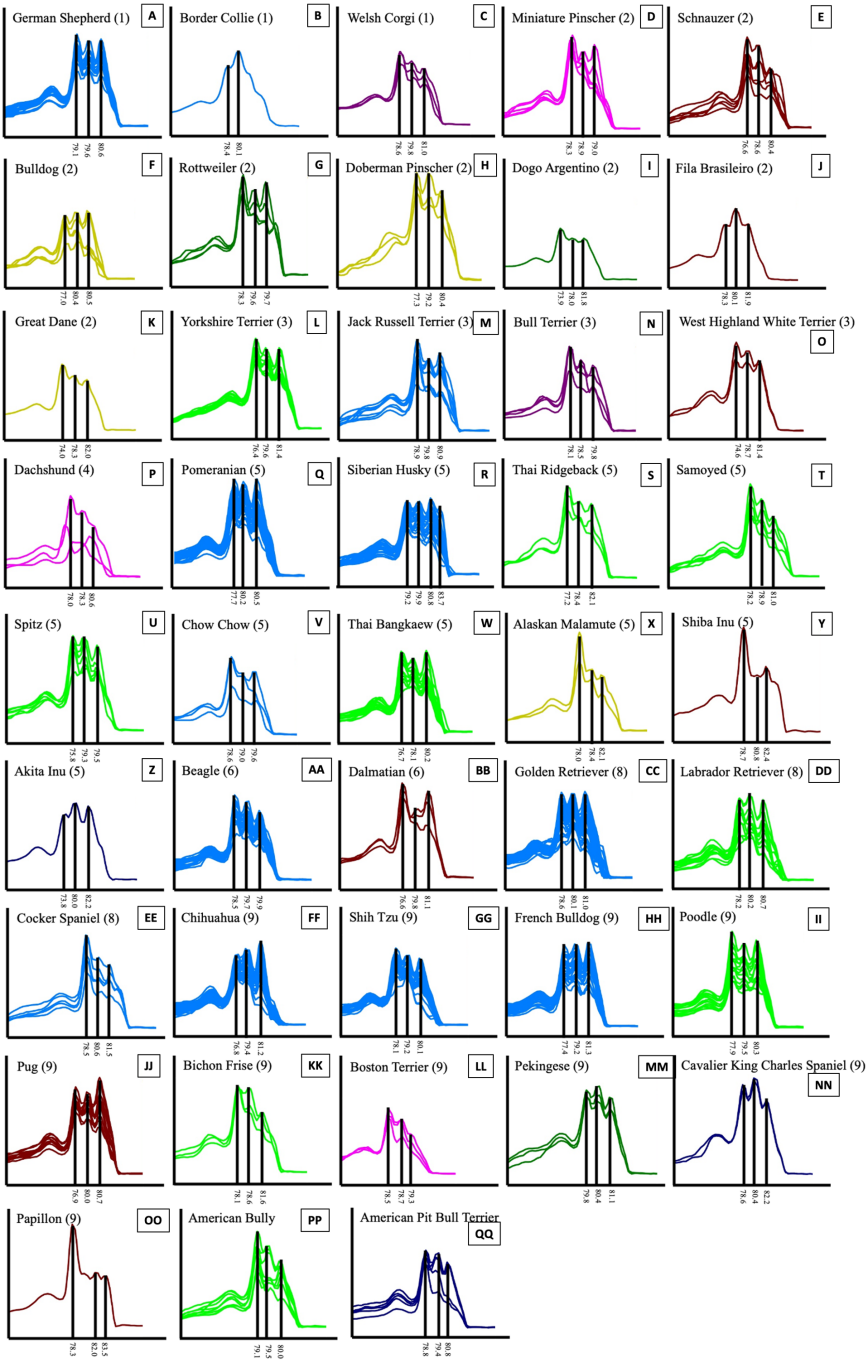
HRM derivative melting curve from 43 different breeds of dogs based on data of UBC823 and dog groups classified by FCI nomenclature. The numbers shown in brackets represent the FCI nomenclature group. German Shepherd (A), Border Collie (B), Welsh Corgi (C), Miniature Pinscher (D), Schnauzer (E), Bulldog (F), Rottweiler (G), Doberman Pinscher (H), Dogo Argentino (I), Fila Brasileiro (J), Great Dane (K), Yorkshire Terrier (L), Jack Russell Terrier (M), Bull Terrier (N), West Highland White Terrier (O), Dachshund (P), Pomeranian (Q), Siberian Husky (R), Thai Ridgeback (S), Samoyed (T), Spitz (U), Chow Chow (V), Thai Bangkaew (W), Alaskan Malamute (X), Shiba Inu (Y), Akita Inu (Z), Beagle (AA), Dalmatian (BB), Golden Retriever (CC), Labrador Retriever (DD), Cocker Spaniel (EE), Chihuahua (FF), Shih Tzu (GG), French Bulldog (HH), Poodle (II), Pug (JJ), Bichon Frise (KK), Boston Terrier (LL), Pekingese (MM), Cavalier King Charles Spaniel (NN), Papillon (OO), American Bully (PP) and American Pit Bull Terrier (QQ).

The results of the melting profiles of 43 dog breeds by ISSR-HRM using 10 primers revealed that the number of distinguishable dog breeds of each primer ranged from 13 to 33 breeds out of a total of 43 breeds (31–78.6%), while UBC827 reported the highest level of discrimination. UBC827 seemed to present indistinguishable melting curve patterns for the Chow Chow, Siberian Husky, Shiba Inu, Thai Ridgeback and Yorkshire Terrier breeds. Moreover, the Chihuahua, Golden Retriever and Pomeranian breeds were grouped together along with the Bichon Frise and Shih Tzu, Beagle and French Bulldog, Welsh Corgi and Poodle breeds. Notably, UBC826 could effectively discriminate between breeds to a value of 76.2% of breed resolution, but it could not distinguish between the Siberian Husky, Pug, Shih Tzu and Spitz breeds. Six other groups that displayed related melting curve patterns were the Bull Terrier, American Bully and Doberman Pinscher, Akita Inu and Great Dane, Alaskan Malamute and Chow Chow, Cavalier King Charles Spaniel and West Highland White Terrier, Papillon and Shiba Inu breeds, along with the Thai Bangkaew and Poodle breeds. Meanwhile, the least effective markers for dog breed discrimination were UBC817 and UBC825, which displayed 38.1% and 31% of breed resolution, respectively (Fig. 4). However, UBC825 could distinguish between certain specific breeds, such as the Dogo Argentino breed, from other similar breeds according to a unique melting shape. Although UBC827 maker analysis yielded a nearly 80% degree of breed discrimination, some dog breeds showed similar patterns of melting shape regardless of their morphology and conformation with other breeds such as Chihuahuas, Golden Retrievers and Pomeranians. As can be seen in Fig. 5, some other dog breeds from the same group according to FCI nomenclature were grouped together based on their similar melting shape. For instance, the Spitz-like group was comprised of the Chow Chow, Siberian Husky, Shiba Inu and Thai Ridgeback breeds. Notably, the Shiba Inu breed generated an isolated melting curve using many ISSR markers such as UBC809 UBC825, UBC826, UBC835, UBC844 and UBC848, along with the Chow Chow breed which initiated a solitary melting curve using UBC817 UBC823 and UBC826. Additionally, in the Siberian Husky group, UBC823, UBC825 and UBC827 markers were used to specifically identify them. Shih Tzu and Bichon Frise breeds displayed the same unique graph pattern when analyzed by UBC827. However, the Bichon Frise group displayed a similar melting pattern to the Shih Tzu group, but these two could be further distinguished from each other using UBC826. Likewise, the Welsh Corgi breed could be distinguished by UBC826. This meant that the Poodle breed displayed the same graph pattern for UBC818 or UBC827. The Alaskan Malamute revealed a similar melting curve as the Chow Chow when analyzed by UBC826. An individual melting graph pattern was observed when we utilized the UBC827 marker. This was a similar outcome to that of Papillon, which displayed a similar graph pattern to Shibu Inu when being analyzed by UBC826. As a result, HRM derivative melting curves could identify 43 dog breeds with a 100% success rate when considering similar melting shapes or patterns from a single primer. This outcome was achieved even when there were different melting shapes or patterns obtained from the other primers such as with five combined or two combined ISSR primers. These are UBC818 and UBC826, UBC818 and UBC827, UBC826 and UBC827, UBC826 and UBC848 and a combination of UBC827 and UBC835 obtained from 45 pairs of the combined ISSR primers (Fig. 5). A combination of UBC817 and UBC825 gave the lowest percent of breed discrimination at 41.86% followed by a combination of UBC825 and 835 and UBC825 and 844 at 46.51%. Additionally, every combination of UBC826 or UBC827 yielded high values at more than 80% in terms of the percent of breed discrimination.

## Discussion

The highlights of this study are that; (i) ISSR-HRM could be a selective tool for facilitating breed discrimination and (ii) genetic variations, differentiations and genetic structure in different dog breeds that reside in northern Thailand revealed a high degree of diversity. However, in some breeds that include a high number of pedigreed dogs, such as the Bull Terrier, the Cavalier King Charles Spaniel, the West Highland White Terrier and the Alaskan Malamute, a lower degree of genetic diversity was observed than in the other breeds. This can lead to an understanding of how genetic variations are related to genetic diseases, which would be useful for all breeding programs. Ultimately, this would result in the breeding of healthier dogs, which can also help researchers develop an understanding of the genes that cause certain breed-specific behaviors (Parker et al., 2004).

### ISSR-HRM for dog breed identification

Our findings indicate that ISSR-HRM could be used to identify all 43 dog breeds through the use of the results of two ISSR markers, especially those obtained from UBC826 and UBC827 which displayed similar repeated motifs (AC)_5_ for the relevant DNA binding sites. Interestingly, from the screening of 45 primer combinations, the Welsh Corgi breed appeared to be the least discriminated breed with a combination of 21 primers. The origin of the Pembroke Welsh Corgi dog suggests that the breed may have originated from central European (Beauchamp, 2010). On the other hand, the Cardigan Welsh Corgi has been associated with certain influences of Nordic settlers in the region, and this determination complies with the outcome that the Welsh Corgi breed was mostly related to certain European and Nordic dogs such as the Alaskan Malamute, Bichon Frise and Doberman Pinscher breeds (McGreevy, 1999). Additionally, the Dogo Argentino is the first and only Argentinean breed that has been accepted by FCI (Caffaratti et al., 2013). This classification separated this breed from others. Hence, Dogo Argentino was the only breed that could be identified with every primer combination. Some of the other dog breeds listed in the same group according to FCI classification were grouped together, for instance the Spitz like group and the Chow Chow, Siberian Husky, Shiba Inu and Thai Ridgeback breeds. This indicated that the ISSR-HRM method could be used to classify dog breeds as a group based on genetic conformation, which could also be linked to similar behaviors or morphologies. However, ISSR-HRM in the current study was established to tackle the problem of misclassification among dog breeds without prior pedigree information. Some dog breeds are very different in their morphologies or behaviors, but they present similar melting patterns such as the Chihuahua, Golden Retriever and Pomeranian, Bichon Frise and Shih Tzu, Beagle and French Bulldog or Welsh Corgi and Poodle breeds. One possible explanation might be due to variations in the number of motifs that repeat, which involve the part of the genes that are associated with distinctive phenotypic changes. For example, in domestic dogs, repeat expansion of microsatellite stretches in the *aristaless-like 4 (ALX-4)* and *runt-related transcription factor 2 (RUNX2)* genes is associated with limb and skull morphology, which reveal interesting correlations between longer sequence lengths of *RUNX2* microsatellites and the longer faces of dogs (Fondon 3rd & Garner, 2007; Sears et al., 2007). This was observed in 30 naturally evolving Carnivora species (Abdurakhmonov, 2016). FCI nomenclature can be used to classify breeds based on morphology, breed history and pedigrees. Notably, dog breeds have been observed in America for more than 10,000 years and distributed to East Asia and European with human migrations. This indicated that most breeds share common ancestry or similar genetic backgrounds even though they have very different appearances (Brown et al., 2015; Castroviejo-Fisher et al., 2011; Wang et al., 2016; Wayne & Ostrander, 1999). Moreover, the primary breed types were developed well, indicating selection and segregation of dogs in the absence of formal breed recognition. Notably, a combination of genetic distance relationships and haplotype pattern sharing and selection has been applied to create many unique combinations of modern traits (Parker et al., 2017).

### Genetic variations in several dog breeds

Some of the breeds analyzed in this study are native breeds with their origins and breed standards being established in Thailand; however, only small populations of these breeds are actually being maintained. The impact of breeding practices on the genetic diversity and the level of inbreeding is critical and of great interest for kennel clubs. The Thai Bangkaew dog is a pure-bred dog that is believed to be indigenous to Thailand. This dog population has developed and carried characteristic genetic signatures that are appropriate for living in Thailand. The values of genetic diversity in this study obtained by ISSR markers were higher when compared with other studies that used alternative fingerprinting markers. The results of this study revealed a high degree of heterozygosity in the Thai Bangkaewusing ISSR markers. The expected level of heterozygosity (H_e_) in the Thai Bangkaew (0.954) breed in this study was higher than in the findings of a previous study involving Thai Bangkaew dogs (0.77) (Phavaphutanon & Laopiem, 2011) which used nuclear microsatellite markers. This might be because dogs are now bred more widely in every part of the country, which makes the genetic markings of the Thai Bangkaew breed more diverse. Moreover, other Asian breeds, such as Sapsaree (0.672), Jindo (0.693), Kishu (0.540), Hokkaido (0.482), Akita (0.544), Shiba (0.310), Sakhalin (0.637), Eskimo (0.599), Taiwan (0.624) and Poongsan (0.840), also revealed a lower degree of heterozygosity (Kang et al., 2009; Kim et al., 2001; Phavaphutanon & Laopiem, 2011). In this study, heterozygosity values were higher than in previous studies among the same dog breeds when different markers were used. For example, many studies researched the genetic diversity of the German Shepherd breed. Their H_e_ values were found to have varied from 0.54–0.71 which was lower than in this study. These results were the same for the Golden Retriever, Jack Russell Terrier, Labrador Retriever, Yorkshire Terrier, Rottweiler, Beagle, Shih Tzu, Bulldog, Bull Terrier, Cocker Spaniel, Poodle, Pug, Pomeranian and Siberian Husky included in previous studies (Bigi et al., 2015; Irion et al., 2003; Kang et al., 2009; Kim et al., 2001; Koskinen & Bredbacka, 2000; Leroy et al., 2009; Mellanby et al., 2013; Wijnrocx et al., 2016). A clarification for these results might be that these breeds in Thailand originated from dog breeds of wide genetic diversity and which had experienced a high degree of gene flow among populations. It is also possible that canine microsatellites revealed a high mutation rate. Additionally, it could also be possible that cosmopolitan breeds that were selected originally for performance are now being bred mainly for conformation. Selection for conformation induces high inbreeding, while selection for performance is more appropriate for maintaining genetic diversity (Parra et al., 2008; Pedersen et al., 2013).

### Genetic differentiations in different dog breeds

Shannon’s information index values were higher than in other studies involving domestic animals using ISSR markers. This was the case among cattle, sheep, buffaloes and goats for which the values ranged from 0.18 to 0.75 (Askari, Abadi & Baghizadeh, 2011; Aytekin et al., 2011; Moradi et al., 2014). These values were also higher than those obtained among the captive Asian elephant at 2.145 (Kriangwanich et al., 2018). Most purebred dogs are morphologically distinct and also differ with regard to their behavior and physical properties. Thus, the higher levels of differentiation in dogs are probably due to the lower utilization of crossbreeding in this species than with other species of domestic animals. The percentage of polymorphic bands also showed a very high level of variability of more than 80% in the Pomeranian, Chihuahua, Golden Retriever, Shih Tzu, Siberian Husky, French Bulldog, Beagle, German Shepherd, Poodle, Yorkshire Terrier, Jack Russell Terrier, American Bully, Samoyed, Schnauzer, Spitz, Bulldog and Dachshund breeds. The Shih Tzu breed had the highest value of polymorphic bands which might have been due to the huge range of colors that exist among the population, including various shades of gold, white, brown and black in the Shih Tzu breed. The high value of polymorphic bands also reveals a range of quality in their differing coat types, such as fine, straight and silky. These differences can affect the genetic diversity. Pairwise genetic distance across the population has been high, which might indicate that at present, inbreeding is not a problem among these dog breed populations in northern Thailand. Genetic variations are a basic requirement for animal breeding as well as for genetic improvement. Some dog breeds may originate from a founder of wide genetic diversity, which would indicate that they had experienced genetic flow among populations. Moreover, breeding selection is not limited to only some popular sires or dams, but they have the opportunity to share their genetic material with other populations. Therefore, artificial selection and close breeding are not intense in these select dog breeds (Phavaphutanon & Laopiem, 2011; Wijnrocx et al., 2016). Possible negative Fis values have been found in some high homozygosity breeds. This is true with certain dogs that are common in Thailand, especially among pure bred dogs that have originated from other countries or those that have come from breeders who may draw from multiple sources and origins. Moreover, the breeding programs employed on most farms usually try to retain variations and heterozygosity in an attempt to prevent instances of inbreeding that can lead to inherited disorders among purebred dogs.

### Dog breed population structures

ISSR fingerprints obtained from 10 primers were used to construct a dendrogram by employing the clustering method of the neighbor-joining algorithm. It indicated that all 33 breeds fell into three main groups. Origin might have had a significant effect on genetic variations in this study according to the phylogenetic dendrogram and structure. Notably, the breeds of French Bulldog and Poodle originated from France. This might be the reason why they formed a cluster that was separate from the other breeds. Likewise, a group comprised of German Shepherds and Doberman Pinschers was known to originate from Germany and a group of Pug and Pekingese dogs was known to originate from China. Moreover, geographic origin plays an important role in shaping genetic clusters. According to the dog breed dendrogram results, most Asian dogs share a close genetic relationship with each other. For instance, the Spitz, Thai Ridgeback and Chow Chow breeds, which are considered Asian breeds, are actually all related to the Spitz and are in the same branch as the Shih Tzu, Pekingese and Pug breeds which were all grouped together. Additionally, the results of each significant branching were in clear agreement with the known history of the designated breed. For example, Golden and Labrador Retrievers were found to be very close to each other, which was likely because they are linked to a common historical ancestor (Lesser Newfoundland) and geographic origin (United Kingdom). The American Bully is considered a half-blood breed of the American Pit bull Terrier and other Bulldog breeds, which explains why these breeds were found to be genetically related. Apart from that, dogs that were commonly bred for performance are now being bred mainly for a number of other characteristics. This might account for the high number of hunting dog breeds including Dalmatians, Beagles and Dachshunds, which were all grouped in the same branch (Colie, 2015; Drury, 1903; Levine & Poray-Wybranowska, 2016). Furthermore, 15 dog breeds that displayed more than six samples, the Chihuahua, Pomeranian, Golden Retriever, Shih Tzu, German Shepherd, Thai Bangkaew, Beagle, Siberian Husky, French Bulldog, Poodle, Yorkshire Terrier, Labrador Retriever, Pug, Jack Russell Terrier and American Bully breeds, also displayed a high level of genetic diversity according to phylogenetic dendrograms. In this case, every breed fell into 5 to 6 clusters for each group. From the 15 dog breeds that were mentioned above, STRUCTURE analysis was employed and it indicated that some breeds were weakly defined because there was a concern about the small number of animals in those breeds. Moreover, the results revealed differing outcomes from those of a previous study. The Jack Russell Terrier breed was recognized as a more homogenous group in this study. A study by Mellanby et al. (2013) demonstrated that the Jack Russell Terrier breed made up the most diverse group. This was likely because some of the Jack Russell dogs may have been pure bred or bred from Parson Russell Terriers. This group is not recognized as a Kennel Club-registered breed in the United Kingdom. However, a similar variety, the Parson Russell Terrier, is registered in the UK. On the other hand, Parson Russell Terriers are not a popular breed in Thailand; therefore, the opportunity of crossbreeding between the Jack Russell Terrier and the Parson Russell is less likely than in the United Kingdom (Mellanby et al., 2013). The breed of Chihuahua was separated into one unique cluster when *K* = 4. This might have been the case because the Chihuahua was the only South American breed that was included in the STRUCTURE analysis in this study. Additionally, Spitz related breeds, like the Pomeranian and Spitz, were grouped together with *K* = 4 as well. The Cocker Spaniel breed revealed the greatest level of diversity. This might have been due to the misclassification of the English Cocker Spaniel and the American Cocker Spaniel among veterinarians or because inaccurate information was provided by owners during the sample collection process.

### Limitations of the study

The study on the genetic diversity of canine breeds might be dated; nevertheless, dog breed population diversity in northern Thailand has never been assessed by genetic variations or diversity. However, the limitations of this study include the fact that the number of samples of several breeds was very low. This occurred for a variety of reasons, such as the low degree of popularity of some breeds in Thailand, or the fact that some breeds have only recently been introduced into the country. Moreover, some breeds may be represented by a limited number of samples of no more than 3–4 individuals, while most of them are related. Thus, we had to cut these samples and did not include them in this study. Furthermore, ISSR-HRM is a more convenient method for assessing the melting profile and can be employed to separate dog breeds. Nevertheless, some dog breeds displayed variations in the melting profile. This might have occurred because of the high genetic variations that exist in some of the breeds that are known to populate northern Thailand. Consequently, this would have likely affected the melting profile or pattern.

## Conclusion

The ISSR-HRM analyses revealed discrepancies of the melting profiles among different dog breeds. The high power of discrimination could be augmented using a combination of two ISSR primers. Our findings demonstrated that the combination ISSR markers obtained from the HRM techniques could be an optional tool for breed identification. Moreover, the results revealed a high diversity and very low level of inbreeding within and between different dog breeds. Regardless, according to the results, the incorporation of ISSR marker analysis with other informative genetic markers should greatly improve the accuracy of inter-breed genetic distance and intra-breed diversity estimates in animals. Therefore, effective and proper breeding management schemes in these dog breeds is advisable in order to avoid significant increases in incidences of inbreeding and losses in terms of genetic variations.

##  Supplemental Information

10.7717/peerj.10215/supp-1 Supplemental Information 1 Number of samples, name of different dog breeds with abbreviation indicated as ”Pop” Information of 2 bands for genetic variation and differentiation analysis among different dog breeds and also dog breed population structure and phylogenetic dendrograms were presented in each 10 ISSR primer, UBC809, UBC817, UBC818, UBC823, UBC825, UBC826, UBC827, UBC835, UBC844 and UBC848 as base pair.

10.7717/peerj.10215/supp-2 Supplemental Information 2 A representative gel of Chihuahua using ISSR primer UBC817 The first lane represents 100 bp ladder, while lanes 2-14 represent individual samples and lane 15 represents the negative control. The photo-image (A) depicts the representative gel and the image below (B) displays the scored bands in animated format.

10.7717/peerj.10215/supp-3 Supplemental Information 3 Number of bands and band size range from each primer from 43 different dog breeds

10.7717/peerj.10215/supp-4 Supplemental Information 4 Pairwise population matrix of Nei’s Genetic Distance are below the diagonal and Pairwise population Fst values, above the diagonal, in overall population of different dog

## Additional Information and Declarations

### Data Availability

The following information was supplied regarding data availability: The raw data is available as a Supplemental File.
