# Supplementary figures and images for: Genetic variations and dog breed identification using inter-simple sequence repeat markers coupled with high resolution melting analysis
Source: PeerJ. 2020 Oct 30;8:e10215. doi: 10.7717/peerj.10215 (PMC7605226; doi:10.7717/peerj.10215)

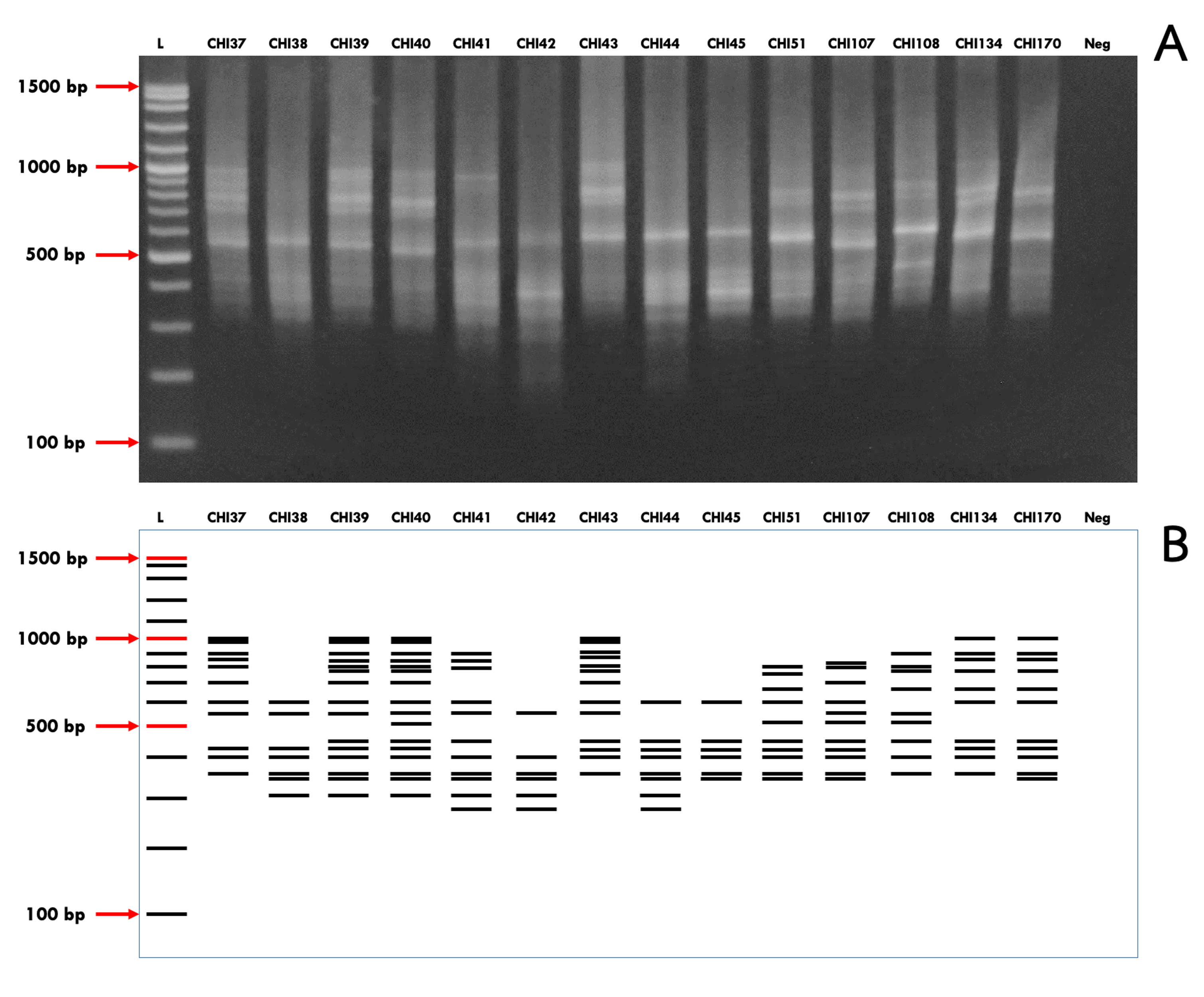

Supplement: Supplemental Information 2 — The first lane represents 100 bp ladder, while lanes 2-14 represent individual samples and lane 15 represents the negative control. The photo-image (A) depicts the representative gel and the image below (B) displays the scored bands in animated format. [file peerj-08-10215-s002.png]
